# Risk factors associated with functional decline in older hospital survivors with acute lower respiratory tract infections: a prospective cohort study

**DOI:** 10.1186/s12877-024-04838-0

**Published:** 2024-02-29

**Authors:** Bingxuan Weng, Jin Jin, Lixue Huang, Xunliang Tong, Wenshu Jiao, Yuanqi Wang, Chuangsen Fang, Mengyuan Wang, Yanming Li

**Affiliations:** Department of Respiratory and Critical Care Medicine, Beijing Hospital, National Center of Gerontology, Institute of Geriatric Medicine, Chinese Academy of Medical Sciences, Beijing, 100730 China

**Keywords:** Physical function, Frailty, Geriatric assessment, Lower respiratory tract infections, The older

## Abstract

**Objective:**

To evaluate the dynamics of basic activity of daily living (BADL) in older patients with acute lower respiratory tract infections (LRTIs) during acute phase and to investigate risk factors associated with decreased physical function at discharge.

**Methods:**

We conducted a prospective cohort study of patients aged 65 years and older who were hospitalized for acute LRTIs between April 15, 2020 and January 15, 2023. All patients received geriatric assessment at admission, including emotion, cognition, frailty, physical function status and so on. The BADL was also evaluated by the Barthel Index (BI) at two weeks before admission by recall (baseline status), at admission and at discharge. Based on the BI grades at baseline and at discharge, patients were classified into two groups: ADL decline and no ADL decline. Multivariable adjusted logistic regression models were used to evaluate the risk factors of decreased physical function.

**Results:**

A total of 364 older survivors with LRTIs were included in the analysis. The median age was 74 years (IQR 61.0–82.0), 231 (62.6%) were male, the median length of stay was 10 days. In the geriatric assessment, 139 patients (38.2%) were classified as frailty, 137 patients (37.6%) experienced insomnia, 60 patients (16.5%) exhibited cognitive impairments, and 37 patients (10.2%) were defined as malnutrition. Additionally, 30 patients (8.2%) dealt with emotional disorders. On average, patients were taking 3 medications, and Charlson Comorbidity Index score was 4. 72 patients (19.8%) had function decline at discharge. In the multivariable analysis, frailty status had an odds ratio of 4.25 (95% CI 1.31–19.26) for decreased physical function and cognitive impairment had an odds ratio of 2.58 (95% CI 1.27–5.19).

**Conclusions:**

About 20% older patients with LRTIs experienced functional decline at discharge. Compared to age, severity of diseases and length of stay, frailty and cognitive impairment performed better at predicting the function decline. The apply of geriatric assessment may contribute to enhance the quality of management and treatment for patients with the older with LRTIs.

## Background

Lower respiratory tract infections (LRTIs) stand as one of the deadliest infectious diseases, ranking fourth among leading causes of death. In 2019, the global incidence of LRTIs reached approximately 6,318.64 cases per 100,000 people, claiming the lives of 2.6 million individuals [1]. Previous researches on this area have paid lots of attention to pathogens, clinical presentations and severity of diseases, largely predominantly disease-driven, overlooking the physiological and pathological characteristics specific to the older. The geriatric assessment (GA) serves as a fundamental approach for evaluating physiological status. The role of GA in predicting adverse outcomes and enhancing prognosis has been well-documented in other acute conditions, such as hip fractures, heart failure [2–6]. However, the dimensional profile of damage and the role of geriatric assessment (GA) in acute LRTIs remain unclear.

According to previous researches, more than one-third of older people who are admitted to hospital experience significant functional decline with reduced ability to perform activities of daily living (ADL). This functional decline has been found to be significantly associated with an increased risk of falls after discharge, reduced independence, hospital readmissions, unplanned admissions to residential aged care homes and post-hospital mortality [7–10]. According to a study, patients who were discharged with new or additional disability in self-care ADL, with high rates of 1-year mortality (41.3%vs 17.8%) and less than one third recovering to their baseline level of function [10]. Hence, maintaining functional independence is more important than mere survival in the older [11]. However, there existed a few studies on functional decline in elderly patients hospitalized for LRTIs.

Since physical function is of significance for the elderly and accurately predicting the prognosis in elderly patients with LRTIs is crucial, as such patients often face decisions about complex therapies and interventions. Therefore, we conducted a prospective observational study to evaluate the physical function and investigate the risk factors of function decline at discharge using GA in the elderly with acute LRTIs.

## Methods

### Study design and participants

This was a prospective, observational, longitudinal cohort study conducted in the Department of Respiratory and Critical Care Medicine, Beijing Hospital, a tertiary care hospital in Beijing that mainly served the older patients. Patients aged 65 years or older who were hospitalized for LRTIs were enrolled consecutively from April 15, 2020 to January 15, 2023. The diagnosis of LRTIs was established when one of the following two conditions was met: 1. The patient has a cough, thick sputum and wet rhonchi in the lungs with any of the following: (1) Fever. (2) Elevated total white blood cell and/or neutrophil count. (3) X-ray or Computed tomography shows inflammatory infiltrative lesions in the lungs. 2. Patients with chronic respiratory diseases in a stable phase (chronic bronchitis, chronic obstructive pulmonary disease, bronchiectasis) following an acute infection with pathogenetic changes or chest radiographs showing significant changes or new lesions compared with admission [12]. Patients were excluded if they were totally dependent for all activities of daily living, deaf, had severe cognitive impairment, had advanced malignant tumors, or had an expected survival of less than 1 year.

The study was approved by the medical ethics committee of Beijing Hospital. Written informed consent were obtained from all participants at the time of admission. (ClinicalTrials.gov ID, ChiCTR2100045574)

### Data collection

Before patients were enrolled in the study, two qualified physicians assessed them to determine their eligibility according to the inclusion and exclusion criteria at the time of admission. Subsequently, a trained nurse initiated the assessment process. We collected demographic characteristics (age, sex, history of alcohol drinking and cigarette smoking), the severity of disease(was evaluated by the WHO Clinical Progression Scale [13], ranging from 0 (not infected) to 10 (dead)) and geriatric assessments (including physical function, body mass index(BMI), cognitive ability, sleep and emotion assessment, frailty, the use of medication, comorbidity and social aspects).

The study outcome was defined as a decline in Barthel Index (BI) grades at baseline (two weeks before hospitalization by recall) and at the time of discharge.

### Geriatric assessments

Prior to initiating the research, a series of three comprehensive training sessions were conducted for the assessors, who were qualified nurses from the department. All participants were assessed by trained nurses at admission. All of the participants were subjected to the following assessments.

**Physical function** was assessed by the activity of daily living (BADL), which was evaluated by Barthel Index, a valid functional measurement, which has good test-retest reliability. BI was based on several daily activities (feeding, bathing, grooming, dressing, bowel and bladder control, toilet use capability, transfer from bed to chair and vice-versa, mobility on level-surfaces, and capability to climb stairs), with a higher score indicating a better functional status. It has five grades: completely independent (100 points), minimally dependent (75–95 points), mildly dependent (50–70 points), severely dependent (20–39 points), and completely dependent (< 20 points) [14]. The self-reported BI two weeks before hospitalization was defined as baseline function status.

**Frailty** was assessed by Fried Frailty Score Measures [15] through five criteria: unintentional weight loss, self-reported exhaustion, weakness or poor handgrip strength, slow walking speed, and low physical activity. Patients were defined as frailty if met 3 or more criteria, pre-frailty if met 1 or 2 criteria, or robust if met none of the criteria.

**Cognitive measurement** was measured by Minimum Mental State Examination (MMSE) [16], Patients were defined as cognitive impairment if their MMSE score was 24 or lower(with a cut-off score of 17 for illiterate individuals, 20 for participants with elementary school education, and 24 for those with middle school education and above).

**Affective measurements** were measured by Patient Health Questionnaire-2 (PHQ-2), Generalized Anxiety Disorder-2 (GAD-2), with a cut off score of 3 [17, 18].

**Sleep disorder** was assessed using the Athens Insomnia Scale (AIS), with a cut-off score of 6 [19].

**Malnutrition** was assessed by body mass index(BMI) < 18.5 kg/m2 to define malnutrition according to European Society of Clinical Nutrition and Metabolism(ESPEN) recommendation [20].

**Comorbidity** was assessed by self-reported and physician-recorded conditions. The Charlson comorbidity Index(CCI) was used to quantify the comorbidity burden.

**Social aspects** were assessed by education, family status, satisfaction with the pension.

### Statistical analysis

As indicated by the Shapiro-Wilk test, continuous variables exhibited a non-normal distribution and were presented as median (interquartile range), while categorical variables were expressed as absolute values (percentages), respectively. According to the difference in the ADL between baseline status and at discharge, all patients were classified into two groups: functional decline group and no functional decline group. For the comparison between both groups, Mann Whitney U test was used for the Continuous variables, and χ2 test or Fisher’s exact test was used for categorical variables as appropriate. The univariate logistic regression model was used to explore the risk factors for decreased physical function, and those variables with a *p* value < 0.05 or clinically significant were included in the multivariate logistic regression. Lastly, in order to test the association between frailty and other variables, correlation was calculated by Kendall’s test and were shown in a heatmap. All statistical analyses were performed using the R software (version 4.2.2). A two-sided *P* value < 0.05 was considered statistically significant.

## Results

### Study population characteristics

**Fig. 1:**
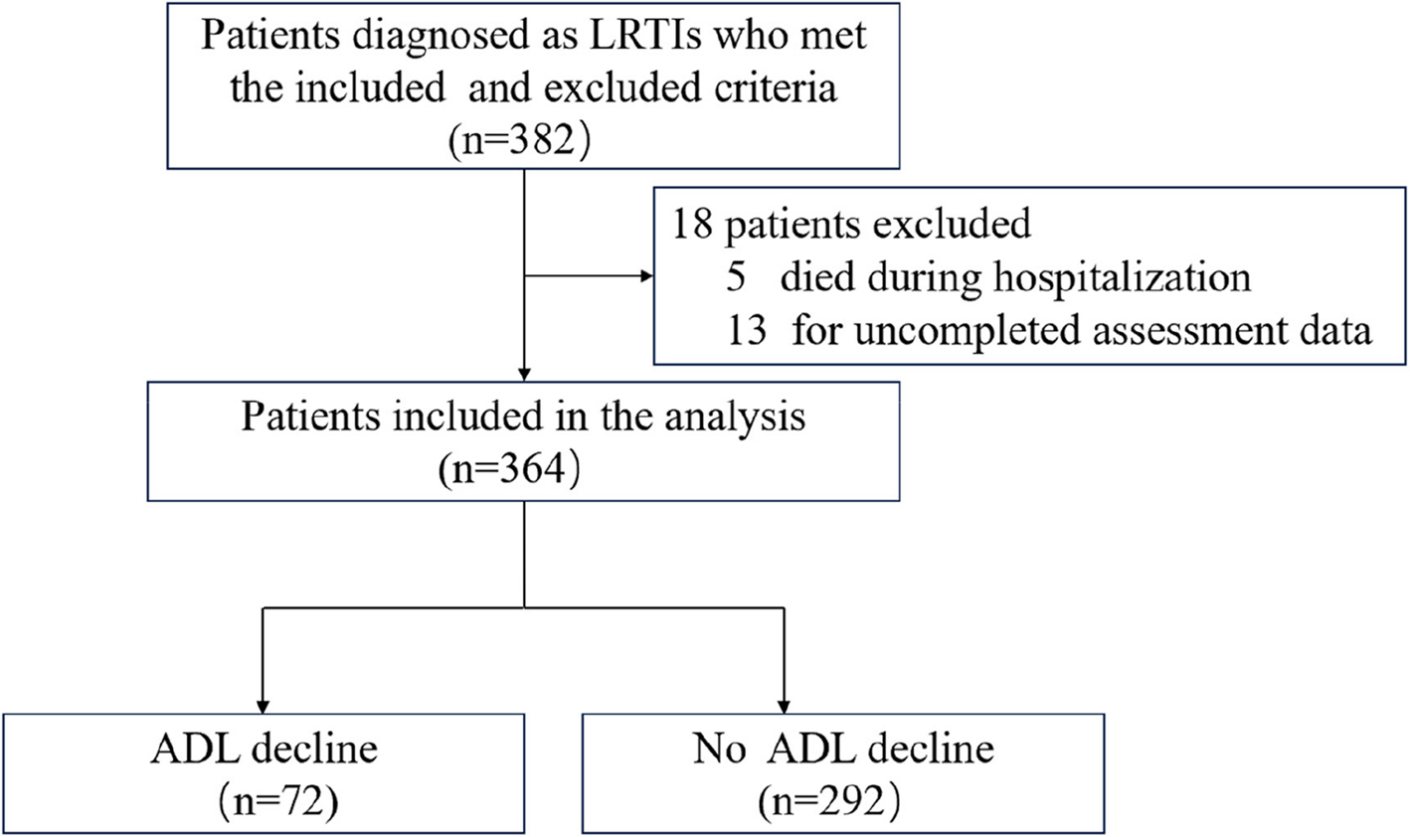
Flow chart of the study

From April 15, 2020 to January 15, 2023, a total of 382 patients were diagnosed with acute LRTIs, of which 13 were excluded due to incomplete assessment data and 5 due to in-hospital death (Fig. 1). Eventually, a total of 364 older survivors with LRTIs were included in the analysis, with a median age of 74 years (interquartile range 69.0 to 82.0), and 62.0% were male. Among them, 221 patients (60.7%) were classified as WHO 4 (hospitalized, no oxygen therapy), 116 patients (31.9%) were classified as WHO 5 (hospitalized, receiving oxygen via mask or nasal prongs), and 27 patients (7.4%) were classified as WHO 6 (hospitalized, receiving oxygen via non-invasive ventilation or high-flow). In the geriatric assessment, frailty (139,38.2%) and insomnia (137,37.6%) were the most commonly impaired dimensions, with cognitive impairments (60, 16.5%) following behind. Additionally, 37(10.2%) patients were defined as malnourished and 30 (8.2%) experienced emotional disorders. The average BMI was 23.50 kg/m^2^, the average CCI score stood at 4, individuals used an average of 3 medications, and the mean ADL score at enrollment was 95.00 (Table 1).

**Table 1:** Characteristics of 364 hospital survivors with LRTIs

	All (*N* = 364)	No ADL decline (*N* = 292)	ADL decline (*N* = 72)	*P* value
**Age, years**	74.0[69.0, 82.0]	74.0 [69.0, 82.0]	76.00 [69.00, 84.00]	0.259
**Sex, n (%)**				0.548
Male	229 (62.0)	181 (62.0)	48 (66.7)	
Female	135 (37.1)	111 (38.0)	24 (33.3)	
**Smoking history, n(%)**				0.364
Never-smoker	203 (55.8)	168 (57.5)	35 (48.6)	
Current-smoker	34 (9.3)	27 (9.2)	7 (9.7)	
Former smoker	127 (34.9)	97 (33.2)	30 (41.7)	
**Drinking history, n(%)**				0.580
Never-drinker	219 (60.2)	177 (60.6)	42 (58.3)	
Current-drinker	79 (21.7)	65 (22.3)	14 (19.4)	
Former drinker	66 (18.1)	50 (17.1)	16 (22.2)	
**Severity of disease during hospitalization, n (%)**				**< 0.001**
WHO 4	221 (60.7)	188 (64.4)	33 (45.8)	
WHO 5	116 (31.9)	89 (30.5)	27 (37.5)	
WHO 6	27 (7.4)	15 (5.1)	12 (16.7)	
**Geriatric assessments**				
**Family status, n(%)**				0.913
Unmarried	1 (0.3)	1 (0.3)	0 (0.0)	
Married	296 (82.5)	239 (82.7)	57 (81.4)	
Divorced	7 (1.9)	6 (2.1)	1 (1.4)	
Widowed	55 (15.3)	43 (14.9)	12 (17.1)	
**Education level, n(%)**				0.623
Illiteracy	11 (3.0)	10 (3.4)	1 (1.4)	
Primary school	33 (9.1)	24 (8.2)	9 (12.5)	
Middle school	191 (52.5)	154 (52.7)	37 (51.4)	
College or higher	129 (35.4)	104 (35.6)	25 (34.7)	
**Attitude towards pension, n(%)**				0.765
No income	6 (1.6)	4 (1.4)	2 (2.8)	
Satisfied	240 (65.9)	193 (66.1)	47 (65.3)	
Generally satisfied	91 (25.0)	74 (25.3)	17 (23.6)	
Unsatisfied	27 (7.4)	21 (7.2)	6 (8.3)	
**Body mass index (kg/m**^**2**^**)**	23.50 [20.80, 25.90]	23.80 [20.90, 25.90]	23.35 [20.78, 26.02]	0.373
**Malnutrition, n (%)**	37(10.2%)	30(10.3%)	7(9.9%)	1.000
**Emotional disorders, n (%)**	30 (8.2)	22 (7.5)	8 (11.1)	0.454
**Sleep disorders, n (%)**	137 (37.6)	106 (36.3)	31(43.1)	0.568
**Cognitive impairment, n (%)**	60 (16.5)	33 (11.3)	33 (31.7)	< 0.001*
**Frailty assessment, n (%)**				< 0.001*
Robust	60 (16.5)	57 (19.5)	3 (4.2)	
Prefrailty	165 (45.3)	142 (48.6)	23 (31.9)	
Frailty	139 (38.2)	93 (31.8)	46 (63.9)	
**Charlson comorbidity index**	4.00 [3.00, 6.00]	4.00 [3.00, 5.00]	5.00 [3.00, 6.00]	**0.016**
**Number of medications**	3.00 [2.00, 6.00]	3.00 [2.00, 6.00]	4.00 [3.00, 6.00]	**0.047**
**Functional assessment**				
BADL at baseline	100.00 [90.00, 100.00]	95.00 [90.00, 100.00]	100.00 [90.00, 100.00]	**0.206**
BADL at admission	95.00 [80.00, 100.00]	95.00 [90.00, 100.00]	80.00 [63.75, 100.00]	< 0.001*
**Length of stay, day**	10.00 [7.00, 14.00]	10.00 [7.00, 13.00]	12.00 [8.75, 16.50]	**0.001**
**Laboratory test**				
White cell count, 10^9^/L	6.84 [5.36, 8.88]	6.86 [5.39, 8.75]	6.36 [5.30, 9.62]	0.629
Neutrophil count, 10^9^/L	4.49 [3.33, 6.34]	4.43 [3.29, 6.02]	4.76 [3.35, 7.12]	0.253
Lymphocyte count, 10^9^/L	1.35 [1.01, 1.70]	1.38 [1.05, 1.76]	1.20 [0.85, 1.52]	**0.004**
Hemoglobin, g/L	121.00 [109.00, 133.00]	122.00 [111.00, 134.00]	118.50 [105.75, 129.50]	0.016*
Albumin, g/L	36.00 [33.00, 39.00]	36.02 [34.00, 39.00]	34.00 [31.00, 37.25]	< 0.001*
Creatinine, mmol/L	71.00 [60.00, 85.00]	72.00 [60.00, 85.50]	70.00 [62.00, 83.00]	0.727
Alanine aminotransferase, U/L	16.00 [11.00, 25.00]	16.00 [11.00, 25.00]	16.00 [11.00, 24.80]	0.960
Aspartate transaminase, U/L	19.00 [15.00, 25.09]	19.00 [15.00, 25.09]	18.50 [15.00, 26.25]	0.930

Data are median(interquartile range) or n (%). WHO 4 means hospitalized but no oxygen therapy. WHO 5 means hospitalized, oxygen by nasal or mask prongs. WHO 6 means hospitalized, oxygen by NIV or high flow

### The functional status assessments

**Fig. 2:**
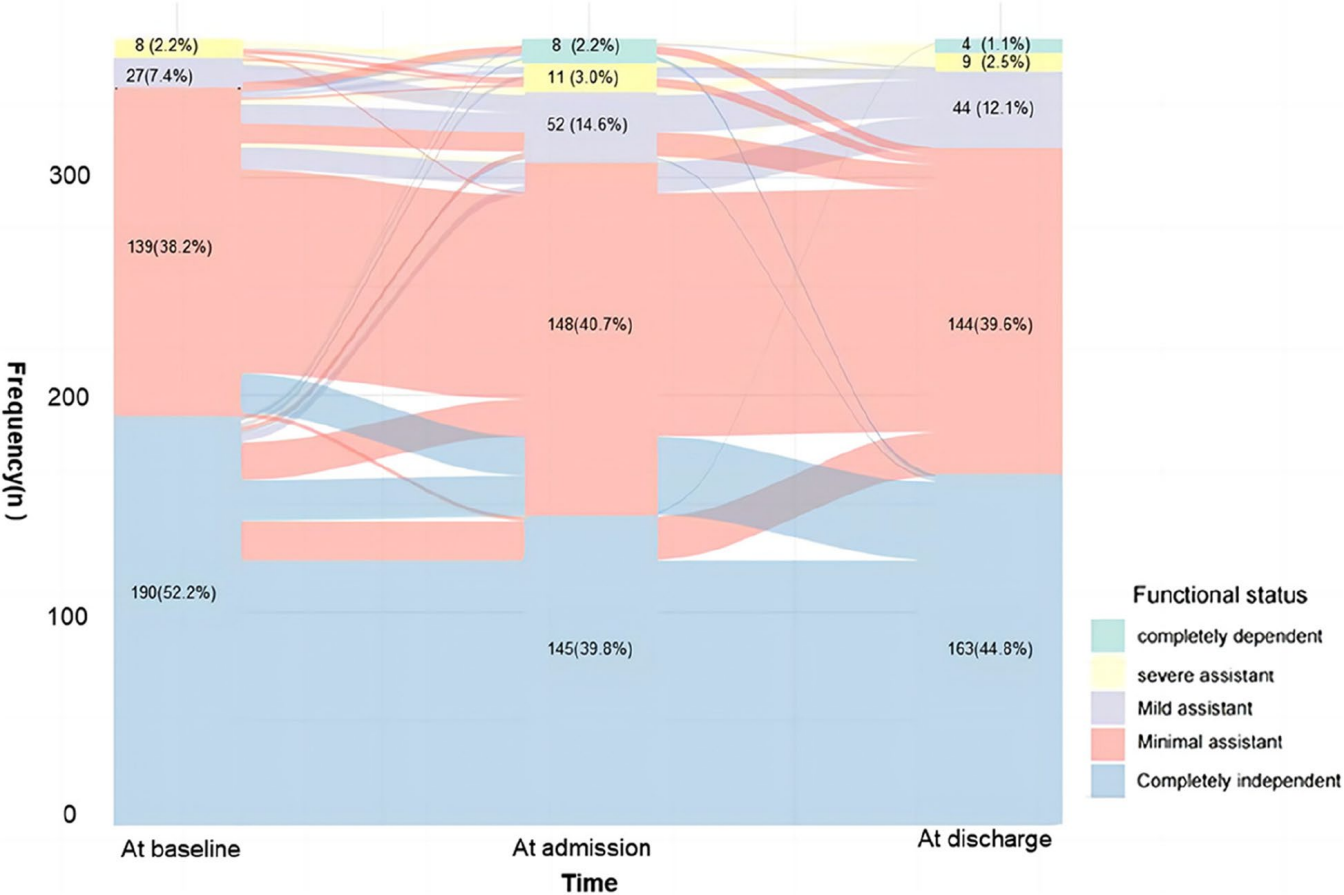
The trajectory of functional status

Two hundred twenty-nine patients consistently maintained their functional status. Seventy-two experienced persistent functional decline. Thirty-four patients initially experienced a decline but subsequently recovered. Among them, 20 patients transitioned from being completely dependent to having functional impairment and then back to complete dependency, 14 progressed from minimal functional impairment to either mild or severe functional impairment, and then reverted to minimal functional impairment. Moreover, 34 patients showed an improvement in their functional status (Fig. 2).

### Factors associated with functional decline at discharge

The results of the univariate analysis of indicate that ADL decline group exhibited significantly association between disease severity scores (WHO6; OR 4.56, 95%CI 1.93–10.62), frailty(OR 9.40, 95%CI (3.24–39.95)), cognitive impairment (OR 4.71, 95%CI 2.58–8.59), CCI (OR 1.12, 95%CI 1.01–1.23), length of stay(OR 1.08, 95%CI 1.04–1.13), lymphocyte count (OR 0.60, 95%CI 0.37–0.93), hemoglobin (OR 0.98,95%CI 0.07–0.99), and albumin (OR 0.92,95%CI 0.87–0.97).

**Fig. 3:**
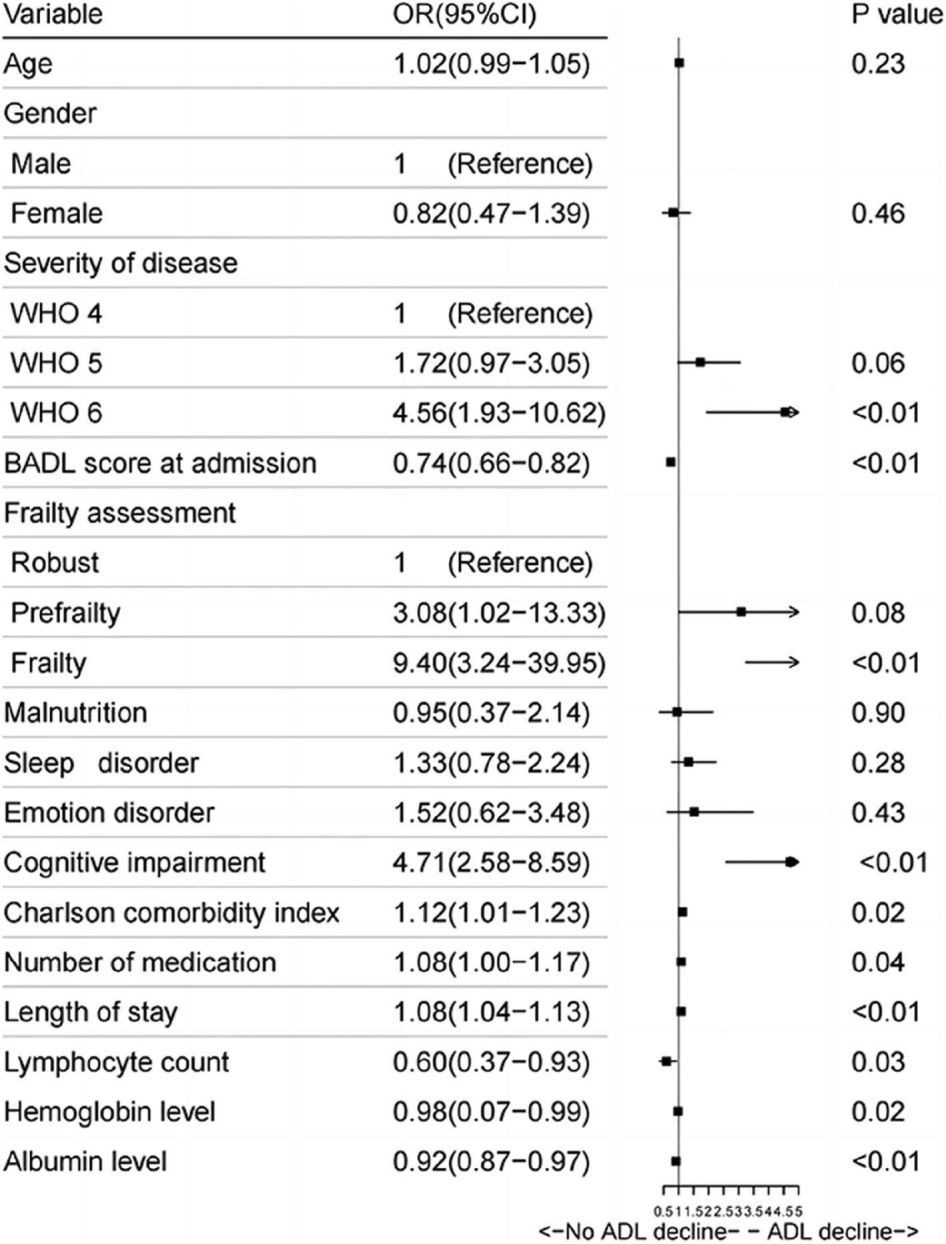
Risk factors for ADL decline at discharge by univariate logistic regression analysis

**Fig. 4:**
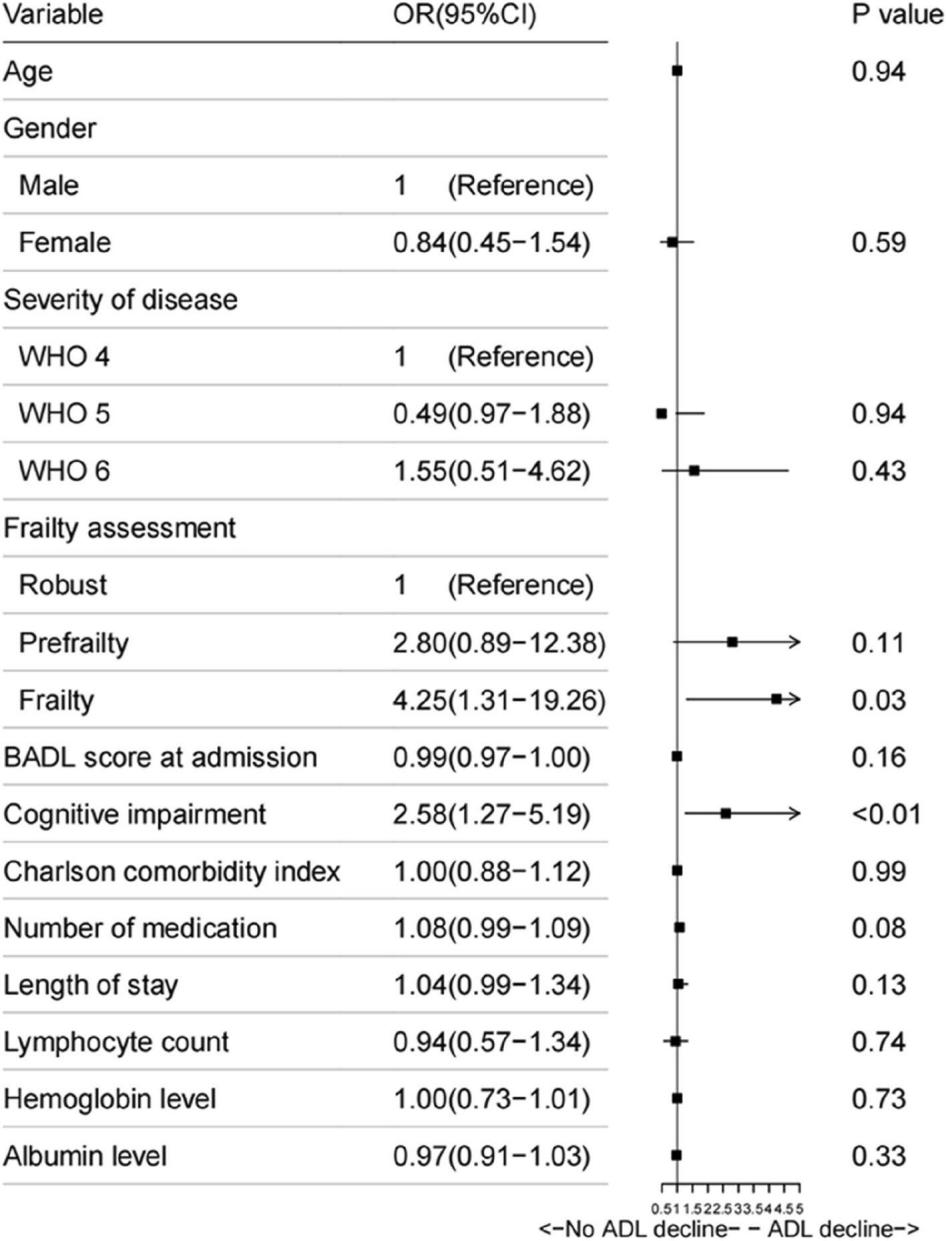
Independent risk factors for ADL decline at discharge by multivariate logistic regression analysis

In the multivariate regression analysis, a significant association was identified between functional decline and frailty (OR 4.25, 95%CI1.31–19.26), cognitive impairment (OR 2.58, 95% CI1.27–5.19) (Figs. 3 and 4).

### The relationship between frailty and other dimensions

**Fig. 5:**
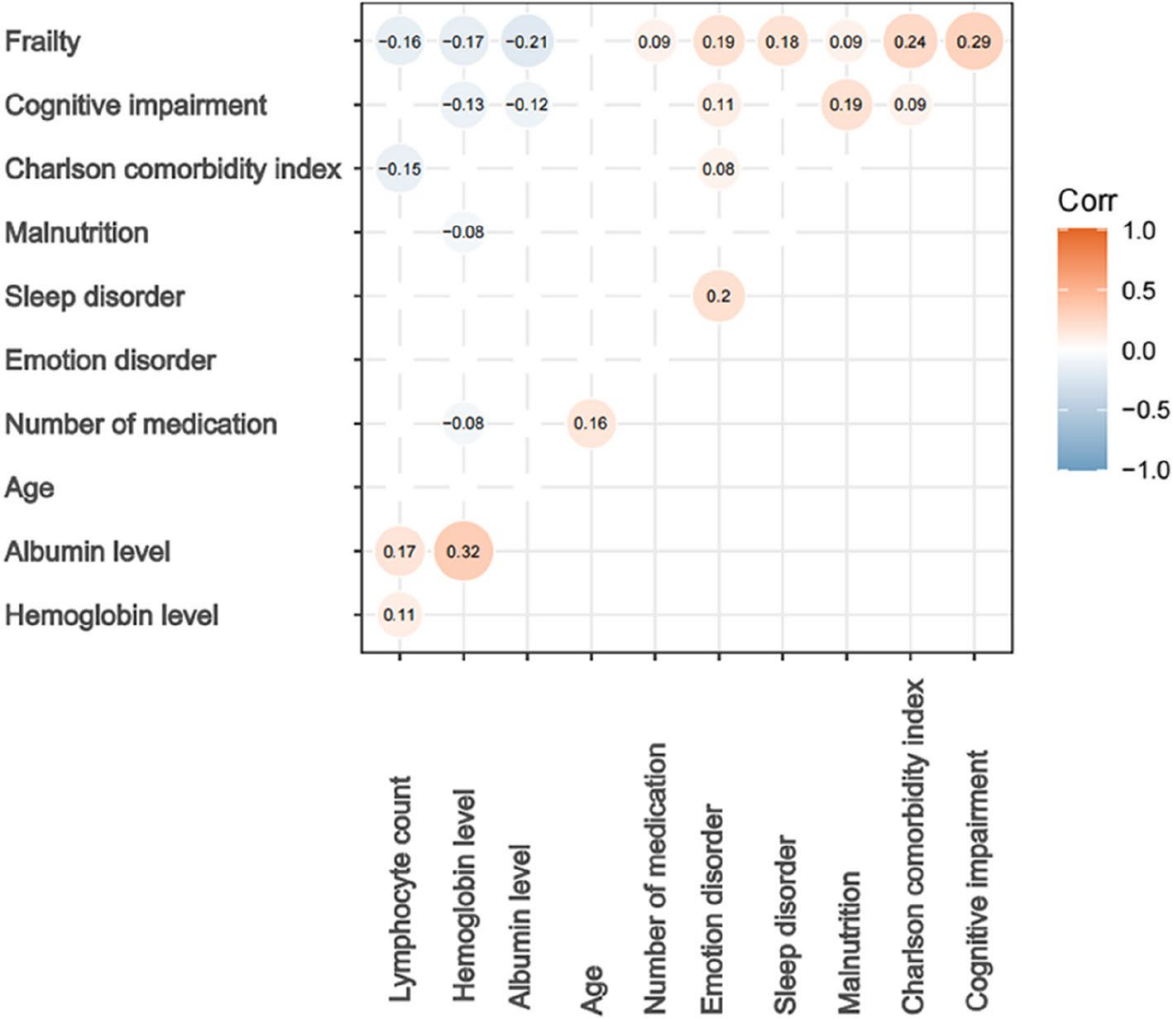
The relationship between frailty and other variables. The correlation between variables was examined using Kendall's test, and *p*-values ＜0.05 were visualized in a circular plot. Correlation coefficients, the Kendall’s tau was annotated

 There were 139 (38.2%) patients classified as frailty, and 165(45.3%) patients were classified as pre-frailty (Table 1). Frailty is related to the higher score of CCI (τ 0.24), higher incidence of emotional (τ 0.19) and sleep disorders (τ 0.21), cognitive impairment (τ 0.29), higher level of neutrophil count (τ 0.14), lower levels of lymphocytes (τ -0.15), hemoglobin (τ -0.17), and albumin (τ -0.21) (Fig. 5).

## Discussion

To our knowledge, the study was the first study use geriatric assessments in the older patients with LRTIs. In the study, we found most patient has at least one-dimension deficits of the assessments, while frailty and insomnia were the most commonly impaired dimensions. We observed that approximately one-fifth older patients hospitalized with LRTIs experienced a decline in their functional abilities upon discharge. Factors independently associated with functional decline were frailty and cognitive impairment rather than age, severity of diseases and length of stay.

Functional decline during hospitalization has been shown to bring to a broad range of adverse outcomes, including falls, poor quality of life, rehospitalization and even death, so early detection was needed to prevent adverse outcome [21–23]. A few studies have described function decline in the respiratory infectious diseases [9, 24–27]. A previous study also found that about 23% older patients hospitalized with influenza suffered from function decline at discharge [16], which indicated functional decline was common in older patients with LRTIs. Consistent with our study, these research efforts emphasize the importance of functionality. However, those researches mainly prioritized pathogen prevention in maintaining function [26, 27] and only a few studies considered the role of malnutrition or frailty in predicting functional decline [9], which were far from capturing the full spectrum of physiological and pathological conditions in elderly individuals.

Our study found that it was not age but rather frailty, that was correlated with the functional decline. Frailty refers to a state of decreased physiologic, functional and cognitive reserve, which increases vulnerability to new health stressors [28]. In our study, approximately 39% of patients exhibited physical frailty, which is consistent with findings from previous research. To the best of our knowledge, limited studies evaluated frailty in the elderly population with acute LRTIs. The prevalence of frailty in these studies ranged from 39.8–54% [11, 29, 30]. In our study, we further founded that frailty was linked with a higher burden of comorbidities, an elevated prevalence of emotional and sleep disorders, cognitive impairment, as well as reduced levels of essential biological markers including lymphocytes, hemoglobin, and albumin. These findings are in alignment with prior research that revealed a correlation between acute functional decline in pneumonia patients and decreased lymphocytes, hemoglobin, and albumin levels [9]. It is plausible that these factors act as intermediary variables between frailty and functional decline. However, the exact causal relationship between these factors and frailty remains unclear, further research is required to validate this hypothesis. Moreover, it is worth noting that frailty is potentially reversible and functional decline may be a preventable disability [31]. Through geriatric assessments, controlling comorbidities, improving emotional and sleep conditions, and enhancing nutritional status, it may be possible to reverse it .

Cognitive impairment, an increasingly recognized as an important and common comorbidity of elderly patients, was also associated with functional decline at discharge. While prior studies have investigated the link between cognitive impairment and physical function in in various contexts, the available evidence on this relationship within the context of LRTIs is somewhat limited. A study based on the UK Biobank database have shown that mortality from LRTIs fell as cognitive ability increase, but without analyzing the connection between cognitive impairments and functional decline [32]. A previous research reported that participants with cognitive impairment had 1.97 times greater odds for IADL disability (CI 1.74–2.23) and 1.26 times greater odds for ADL disability decline (CI 1.05–1.51) among aging Americans [33]. In our study, the prevalence of cognitive impairment in our study was 17.3%, and the group with cognitive impairment had a 2.58-fold higher odds ratio for functional decline compared to the group without cognitive impairment. This result was especially interesting because physical capacity and cognitive function might influence each other in this population; thus, clinical assessment should be implemented. According to previous researches, engaging in aerobic exercise, mental activity, and social interaction may potentially reduce the risk of further cognitive decline [34]. These interventions underscore the importance of early recognition and support for individuals experiencing cognitive impairment.

To our knowledge, our study was the first study to use comprehensive geriatric assessment in the older patients hospitalized with acute LRTIs. We identified frailty and cognitive impairment as risk factors for functional decline, rather than age, severity of disease, length of stay. Besides, we also found that although not all components of the geriatric assessment were directly associated with functional decline, they were linked to frailty to some extent. Therefore, our study emphasizes the critical role of comprehensive geriatric assessment during the acute phase of the respiratory infectious diseases, which may contribute to enhance the quality of management and treatment for patients with the older with LRTIs.

### Limitations

Some limitations of this study should be noted. First, our findings were based on data from a single tertiary care center, which may limit generalizability. Secondly, the basic function status was assessed by recall, which may lead to recall bias, but according to previous studies, functional status 2 weeks prior to disease onset may better reflect baseline function, and this bias may be difficult to avoid. Finally, our observation of functional status up to the time of discharge, long-term follow-up of such patients is needed in the future.

## Conclusions

Functional decline is a common occurrence for older adult hospitalized with LRTIs. As parts of geriatric assessments, compared to age and disease severity, length of stay, frailty and cognitive impairment performed better at predicting the function decline. Therefore, the role of geriatric assessment should be emphasized in acute conditions like elderly infections.

## Data Availability

The original contributions presented in the study are included in the article, further inquiries can be directed to the corresponding author.

## Abbreviations

LRTIs: Lower respiratory tract infections
BADL: Basic activity of daily living
BI: Barthel Index
GA: Geriatric assessment
PHQ-2: Patient Health Questionnaire-2
GAD-2: Generalized Anxiety Disorder-2
MMSE: Minimum Mental State Examination
AIS: Athens insomnia scale
CCI: Charlson Comorbidity Index
